# Community transmission of type 2 poliovirus after cessation of trivalent oral polio vaccine in Bangladesh: an open-label cluster-randomised trial and modelling study

**DOI:** 10.1016/S1473-3099(17)30358-4

**Published:** 2017-10

**Authors:** Mami Taniuchi, Michael Famulare, Khalequ Zaman, Md Jashim Uddin, Alexander M Upfill-Brown, Tahmina Ahmed, Parimalendu Saha, Rashidul Haque, Ananda S Bandyopadhyay, John F Modlin, James A Platts-Mills, Eric R Houpt, Mohammed Yunus, William A Petri

**Affiliations:** a Division of Infectious Diseases and International Health, University of Virginia, Charlottesville, VA, USA; b Institute for Disease Modeling, Global Good, Intellectual Ventures, Bellevue, WA, USA; c International Centre for Diarrhoeal Disease Research, Dhaka, Bangladesh; d David Geffen School of Medicine at the University of California, Los Angeles, CA, USA; e Bill & Melinda Gates Foundation, Seattle, WA, USA

## Abstract

**Background:**

Trivalent oral polio vaccine (tOPV) was replaced worldwide from April, 2016, by bivalent types 1 and 3 oral polio vaccine (bOPV) and one dose of inactivated polio vaccine (IPV) where available. The risk of transmission of type 2 poliovirus or Sabin 2 virus on re-introduction or resurgence of type 2 poliovirus after this switch is not understood completely. We aimed to assess the risk of Sabin 2 transmission after a polio vaccination campaign with a monovalent type 2 oral polio vaccine (mOPV2).

**Methods:**

We did an open-label cluster-randomised trial in villages in the Matlab region of Bangladesh. We randomly allocated villages (clusters) to either: tOPV at age 6 weeks, 10 weeks, and 14 weeks; or bOPV at age 6 weeks, 10 weeks, and 14 weeks and either one dose of IPV at age 14 weeks or two doses of IPV at age 14 weeks and 18 weeks. After completion of enrolment, we implemented an mOPV2 vaccination campaign that targeted 40% of children younger than 5 years, regardless of enrolment status. The primary outcome was Sabin 2 incidence in the 10 weeks after the campaign in per-protocol infants who did not receive mOPV2, as assessed by faecal shedding of Sabin 2 by reverse transcriptase quantitative PCR (RT-qPCR). The effect of previous immunity on incidence was also investigated with a dynamical model of poliovirus transmission to observe prevalence and incidence of Sabin 2 virus. This trial is registered at ClinicalTrials.gov, number NCT02477046.

**Findings:**

Between April 30, 2015, and Jan 14, 2016, individuals from 67 villages were enrolled to the study. 22 villages (300 infants) were randomly assigned tOPV, 23 villages (310 infants) were allocated bOPV and one dose of IPV, and 22 villages (329 infants) were assigned bOPV and two doses of IPV. Faecal shedding of Sabin 2 in infants who did not receive the mOPV2 challenge did not differ between children immunised with bOPV and one or two doses of IPV and those who received tOPV (15 of 252 [6%] vs six of 122 [4%]; odds ratio [OR] 1·29, 95% CI 0·45–3·72; p=0·310). However, faecal shedding of Sabin 2 in household contacts was increased significantly with bOPV and one or two doses of IPV compared with tOPV (17 of 751 [2%] vs three of 353 [1%]; OR 3·60, 95% CI 0·82–15·9; p=0·045). Dynamical modelling of within-household incidence showed that immunity in household contacts limited transmission.

**Interpretation:**

In this study, simulating 1 year of tOPV cessation, Sabin 2 transmission was higher in household contacts of mOPV2 recipients in villages receiving bOPV and either one or two doses of IPV, but transmission was not increased in the community as a whole as shown by the non-significant difference in incidence among infants. Dynamical modelling indicates that transmission risk will be higher with more time since cessation.

**Funding:**

Bill & Melinda Gates Foundation.

## Introduction

In anticipation of the eradication of poliomyelitis, WHO convened a committee in 1998, which recommended that vaccination with oral polio vaccine should stop when there was sufficient confidence in global eradication, suitable containment of poliovirus stocks, and sufficient evidence that Sabin vaccine strains from the oral polio vaccine would not transmit indefinitely in the post-vaccination era.^1^ The committee identified substantial gaps in our understanding of the transmissibility of oral polio vaccine strains and their ability to persist in populations with low and waning immunity.

Because wild type 2 poliovirus has been eradicated, and the Sabin 2 vaccine strain causes most circulating vaccine-derived poliovirus cases and roughly a third of all vaccine-associated poliomyelitis cases, WHO recommended in 2015 the removal of Sabin 2 from the trivalent oral polio vaccine (tOPV) used for routine polio immunisation and mass campaigns in more than 100 countries, replacing tOPV with bivalent types 1 and 3 oral polio vaccine (bOPV) with at least one dose of inactivated polio vaccine (IPV).^2^ Global cessation of tOPV took place in April, 2016, with monovalent type 2 oral polio vaccine (mOPV2) stockpiled for outbreak response in the event that transmission of type 2 poliovirus is detected post-cessation.^3^ In addition to the risks of wild poliovirus reintroduction from breaches in vaccine manufacturer or laboratory containment,^4^, ^5^ it is well established that vaccine-derived polioviruses can transmit indefinitely in populations with insufficient immunity. In 2000, the first known circulating vaccine-derived poliovirus outbreak occurred in Haiti and the Dominican Republic.^6^ Retrospective analysis uncovered other vaccine-derived outbreaks in Belarus^7^ and Egypt,^8^ and circulating vaccine-derived poliovirus outbreaks are known to have occurred in 29 countries as of the end of 2015,^9^ and most recently in Nigeria and Pakistan.^9^

**Research in context**

**Evidence before this study**

We searched PubMed for all papers published up to March, 2017, with the terms: (“polio” OR “poliomyelitis” OR “poliovirus”) AND (“communicability” OR “transmission” OR “transmissibility”) AND (“oral”) AND (“vaccine” OR “poliovaccine” OR “Sabin”). This search identified 511 reports, of which only 81 were transmission studies. Findings of studies from the 1950s and 1960s showed that Sabin strains that constitute trivalent oral polio vaccine (tOPV) are transmissible among intimate contacts and within communities, even in the presence of naturally acquired immunity. Since 2001, it is well understood that transmission of oral polio vaccine can persist in under-immunised populations and regain neurovirulence to cause circulating vaccine-derived outbreaks that, in turn, cause paralysis indistinguishable from that caused by wild poliovirus. Findings of clinical trials in which participants were given challenge doses of monovalent type 2 oral polio vaccine (mOPV2) showed that mixed immunisation schedules of bivalent types 1 and 3 oral polio vaccine (bOPV) and inactivated polio vaccine (IPV) provide superior protection from infection relative to IPV alone, but the protection is inferior to that produced by a full course of tOPV. We do not know of any published studies that describe Sabin 2 transmission in the context of mixed tOPV and bOPV and IPV population immunity.

**Added value of this study**

Our study reports novel data at the population level from the first synchronised switch from tOPV to bOPV and IPV routine immunisation in a cluster-randomised trial, observing community transmission of poliovirus in a low-income rural community setting. 9 months later, after immunisation with either tOPV or bOPV and IPV, we implemented a mass vaccination campaign with mOPV2 to 40% of enrolled infants and community participants younger than 5 years as a novel approach to elicit community dynamics of massive exposure to oral polio vaccine in a trial setting. The switch from tOPV to bOPV and IPV facilitated more transmission of Sabin 2 in the first 10 weeks after an mOPV2 campaign and increased the quantity of virus shed in recipients of bOPV and IPV and their contacts who were not directly vaccinated with mOPV2. Adding a second dose of IPV to the end of the routine immunisation schedule did not mitigate these changes.

**Implications of all the available evidence**

Our data confirm historical observations that, on withdrawal from routine use, Sabin 2 will persist only for limited durations in well-immunised populations irrespective of living conditions and socioeconomic status. The risk of epidemic Sabin 2 transmission remains small in well-immunised communities within 1 year of tOPV cessation, but the increased quantity of virus shed in populations with birth cohorts receiving bOPV and IPV relative to those receiving tOPV indicates that the transmission potential of Sabin 2 will increase as the birth cohort receiving bOPV and IPV grows. This risk should be mitigated by maintaining high population immunity with IPV, improved environmental surveillance for vaccine-derived poliovirus transmission, and maintenance of mOPV2 stockpiles and protocols to respond to vaccine-derived poliovirus outbreaks.

In the 1950s and 1960s, transmission experiments with oral polio vaccine showed that the Sabin strains are transmitted easily among close contacts and within small communities, and that Sabin 2 is the most transmissible of the three strains.^10^, ^11^ These historical studies were done in southern USA under variable sanitation conditions in the context of natural immunity, and with limited exposure to IPV, and it is not known how representative they are in the post-eradication era.^12^ Widespread tOPV vaccination eliminated type 2 transmission wherever sufficient coverage was achieved. bOPV and at least one dose of IPV provides inferior intestinal immunity to type 2 relative to tOPV,^13^ and the degree to which cross-immunity to type 2 poliovirus^14^ will protect against the increased risk of community transmission of Sabin 2 is not known. It is also unknown whether addition of a second IPV dose will confer additional protection against community transmission.

We estimated the effect of tOPV cessation on community transmission of Sabin 2 after mOPV2 reintroduction in a community with multiple risk factors for enteric disease transmission, including high faecal-oral transmission, low socioeconomic status, and high diarrhoeal disease burden, but high immunisation rates (>95% in our study area; roughly 90% in Bangladesh as a whole).^15^

## Methods

### Study design and participants

We did an open-label cluster-randomised study in rural Matlab, Bangladesh, where the International Centre for Diarrhoeal Disease Research, Bangladesh (icddr,b) has done community-based public health research in the context of a health and demographic surveillance system (HDSS) since 1963. The study was undertaken in the Maternal, Child, and Family Planning intervention area, which consists of 67 villages and is inhabited by roughly 130 000 people, with more than 2600 births in 2012.^15^

We included three types of participants in our study: infants, household contacts, and community participants. Medical officers enrolled infants to participate in stool surveillance if they were aged 6 weeks at the time of their first polio vaccination, their parent or guardian's primary residence at the time of vaccination was a participating village, and written informed consent had been obtained before the infant's first polio vaccination. We excluded infants from enrolment for surveillance if they had known hypersensitivity to any component in the vaccines, uncorrected congenital malformation, or known or suspected immunodeficiency. Irrespective of enrolment for stool surveillance, all infants born during our study and without medical contraindication received the immunisation schedule assigned to their village of residence as part of the routine health services provided by icddr,b in the HDSS service area. For each enrolled infant, we also enrolled the two youngest household contacts among the infant's extended family—as defined by their siblings, caregiver, and anyone sharing a cooking pot (*khana*) with the caregiver—to participate in stool surveillance. We enrolled household contacts if their primary residence was in a participating village and written informed consent was obtained before their infant's first polio vaccination. Finally, community participants were children who received mOPV2 but were not enrolled for stool surveillance. To be a community participant, a child had to be younger than 5 years on Jan 24, 2016, have their primary residence in a participating village, and be randomly assigned from HDSS census data as a potential participant. Also, written informed consent must have been obtained by a field worker or study doctor before mOPV2 administration.

Finally, we selected at random 40% of all children younger than 5 years and living in all villages to be community participants and obtained consent to receive mOPV2 as part of a campaign done 2 weeks after completion of enrolment. From the original enrolled households, we randomly selected a prespecified total of 800 enrolled infants and their household contacts for continued intensive surveillance (appendix p 1).

This study was done according to the guidelines of the Declaration of Helsinki. The protocol was approved by the Research Review Committee (RRC) and Ethical Review Committee (ERC) of the icddr,b and the Institutional Review Board of the University of Virginia.

### Randomisation and masking

All villages in the intervention area were randomly allocated to one of three schedules for infant routine immunisation: tOPV at age 6 weeks, 10 weeks, and 14 weeks; bOPV at age 6 weeks, 10 weeks, and 14 weeks and one dose of IPV at age 14 weeks; or bOPV at age 6 weeks, 10 weeks, and 14 weeks and two doses of IPV at age 14 weeks and 18 weeks. No supplemental polio immunisation activities took place in Bangladesh during the study period. For the cluster randomisation, we used constrained randomisation to choose 22 or 23 villages per treatment group, such that the expected total population sizes in each group differed by no more than 5%,^16^ and this process was implemented in *R* version 3.2.2 (R Foundation for Statistical Computing, Vienna, Austria). We randomly selected individuals to receive mOPV2 within each treatment group using MATLAB version 2015b (MathWorks, Natick, MA, USA). Randomisation lists were provided to the investigators, with the exception of members of the laboratory team, who were unaware of all aspects of the randomisation in the study; laboratory work was completed before prespecified analysis timepoints. Statistics team members were unaware of laboratory data until the prespecified analysis timepoints. We were not able to mask the field team or participants to vaccine regimen because IPV requires an injection whereas bOPV and tOPV do not.

### Procedures

Infants received either tOPV (Sanofi Pasteur, Lyon, France) or bOPV (BioFarma, Bandung, Indonesia) and one or two doses of intramuscular IPV (Bilthoven Biologicals, Bilthoven, Netherlands) for polio routine immunisation, according to village schedules (table 1). For the mOPV2 campaign after enrolment, one dose of mOPV2 (GlaxoSmithKline, Rixensart, Belgium) was administered. All vaccines were given by icddr,b staff.

**Table 1:** Timepoints for vaccination and stool specimen collection

	**tOPV**	**bOPV and IPV (one dose)**	**bOPV and IPV (two doses)**
**Vaccine regimen**			
tOPV	6, 10, and 14 weeks	..	..
bOPV	..	6, 10, and 14 weeks	6, 10, and 14 weeks
IPV	..	14 weeks	14 and 18 weeks
BCG	6 weeks	6 weeks	6 weeks
Measles	9 and 15 months	9 and 15 months	9 and 15 months
Penta	6, 10, and 14 weeks	6, 10, and 14 weeks	6, 10, and 14 weeks
PCV	6, 10, and 18 weeks	6, 10, and 18 weeks	6, 10, and 18 weeks
RotaTeq*	6, 10, and 14 weeks	6, 10, and 14 weeks	6, 10, and 14 weeks
Rubella	9 months	9 months	9 months
**Stool collection schedule**			
Enrolment in study	Age 6 weeks	Age 6 weeks	Age 6 weeks
Day after first oral polio vaccine†	Age 6 weeks + 1 day	Age 6 weeks + 1 day	Age 6 weeks + 1 day
After completion of polio vaccine regimen	Age 18 weeks	Age 18 weeks	Age 18 weeks
Day before mOPV2 campaign	Week 0 of mOPV2 campaign	Week 0 of mOPV2 campaign	Week 0 of mOPV2 campaign
Weekly after mOPV2 campaign (every 7 days)	Weeks 1–10 of mOPV2 campaign	Weeks 1–10 of mOPV2 campaign	Weeks 1–10 of mOPV2 campaign
Monthly, 10 weeks after mOPV2 campaign (every 28 days)	Weeks 14, 18, and 22 of mOPV2 campaign	Weeks 14, 18, and 22 of mOPV2 campaign	Weeks 14, 18, and 22 of mOPV2 campaign

Data are age of vaccinated infant for vaccine regimen; and age of infant or time in mOPV2 campaign for stool collection schedule. BCG=bacillus Calmette-Guérin vaccine against tuberculosis. bOPV=bivalent types 1 and 3 oral polio vaccine. IPV=inactivated polio vaccine. mOPV2=monovalent type 2 oral polio vaccine. PCV=pneumococcal conjugate vaccine. Penta=pentavalent vaccine for diphtheria, tetanus, whooping cough, hepatitis B, and *Haemophilus influenzae* type B. RotaTeq=pentavalent oral vaccine against rotavirus. tOPV=trivalent oral polio vaccine.

* Roughly 100 enrolled infants did not receive all or part of the RotaTeq series because of supply issues.

† Stool specimens gathered only from enrolled infants.

At the time of enrolment, we handed out stool containers with instructions to obtain stool from the infant before the first polio vaccination (age 6 weeks), 1 day after the first vaccination, and at age 18 weeks (table 1). For enrolled household contacts, stool samples were collected on the same day as the corresponding infant's stools at enrolment and at age 18 weeks. For the subset of individuals randomly selected for intensive weekly surveillance around the mOPV2 campaign, stool samples were collected from infants and household contacts immediately before the mOPV2 campaign (week 0), then every week for 10 weeks (weeks 1–10), then monthly thereafter for 3 months (weeks 14, 18, and 22; table 1). The mOPV2 campaign and subsequent stool collections were organised by village block,^15^ with block A visited on Mondays, block B on Tuesdays, block C on Wednesdays, and block D on Thursdays.

For each village, we collected stool samples in stool containers; 4·C cold chain was maintained and samples were delivered to the laboratory at icddr,b in Matlab within 6 h of collection. Stool specimens were divided into aliquots on receipt and then stored at −80·C. We put samples into batches and shipped them on dry ice to icddr,b in Dhaka. We extracted total nucleic acid from stool samples and used multiplex quantitative reverse transcriptase PCR (qRT-PCR) to detect Sabin viruses and an extrinsic control, bacteriophage MS2, as described previously.^17^ Briefly, we extracted total nucleic acid from 200 mg of stool using the QIAamp Fast DNA Stool mini kit (Qiagen, Hilden Germany). We added two extrinsic controls—PhHV (phocine herpesvirus) and bacteriophage MS2—to the lysis buffer to monitor extraction and amplication efficiency.^18^, ^19^ The specimen underwent bead beating and was then extracted according to the manufacturer's manual. We stored total nucleic acid at −80·C until PCR testing. We converted threshold cycles (Ct) to viral copy numbers^17^ and calculated the median shedding index as the proportion shedding after mOPV2 challenge multiplied by the median shedding duration of those who shed multiplied by the median concentration shed per sample.

### Outcomes

We assessed outcomes in the per-protocol population, which we defined as all infants aged 18 weeks or older at the time of the mOPV2 campaign and their household contacts, excluding those who were shedding Sabin 2 immediately before the mOPV2 campaign. We restricted our analysis to the per-protocol population to maintain balance across trial arms while minimising confounding from shedding in young infants who had not completed their tOPV schedules.

The primary outcome was the incidence of transmission-acquired Sabin 2 infections in enrolled infants during the first 10 weeks after the mOPV2 campaign. We defined incidence as the proportion of infants in the per-protocol cohort who did not receive mOPV2, were negative for type 2 poliovirus at the time of mOPV2 challenge, and provided at least one stool sample positive for Sabin 2 at any point during the 10 weeks after, adjusted for within-village correlation. The primary hypothesis was that incidence would be higher in infants treated with bOPV and either one or two doses of IPV relative to those treated with tOPV. The two groups treated with bOPV and one or two doses of IPV were combined in the preplanned analysis of the primary objective because any potential difference between the two IPV-containing treatments was expected to be small by comparison with the difference between bOPV and tOPV.^13^

Secondary objectives were to test the hypothesis that two doses of IPV would reduce transmission in infants treated also with bOPV relative to those treated with bOPV and one dose of IPV, and to measure the intestinal immunogenicity (incidence of shedding) in individuals who directly received mOPV2. Descriptive outcomes included incidence of Sabin 2 transmission in household contacts and the average concentrations of poliovirus shed (viral copy number per g of stool) in each trial arm. All adverse events were reported by fieldworkers, investigated by icddr,b doctors, and were recorded with the symptoms, diagnostic results, duration, and evidence of relation to study procedures.

### Statistical analysis

After the mOPV2 campaign, we modelled the incidence of transmission-acquired type 2 poliovirus infections in individuals who did not receive mOPV2 using generalised linear mixed model (GLMM) logistic regression with binomial distribution and logit link function between incidence 10 weeks after the campaign and the covariate treatment arm and a random intercept for each village to account for within-village (intracluster) correlation. We calculated the significance of differences between tOPV and both bOPV and IPV schedules using a one-sided Wald test (α=0·05) for the hypothesis that the combined mean incidence over the first 10 weeks in both bOPV and IPV groups (assuming normal residuals) was higher than the incidence with tOPV. From the power analysis done during the design phase of the study, we expected greater than 99% power to detect the expected increase in Sabin 2 incidence in both bOPV and IPV groups combined, assuming an expected incidence after 10 weeks of 1·5% with tOPV and 22% with both bOPV and IPV schedules combined; we derived expected incidence from simulations using the individual-based Institute for Disease Modeling polio model,^20^ with realistic polio immunity and transmission parameters calibrated to achieve 6·6% shedding in infants aged 6 weeks during a tOPV routine immunisation schedule with 94% coverage of the third dose of polio vaccine.^15^ We also tested the hypothesis that incidence would be lower with bOPV and two doses of IPV than with bOPV and one dose of IPV; based on the modelled difference in intestinal immunity between arms derived from Behrend and colleagues,^21^ we expected 91% power to detect the expected decrease in incidence. We did similar GLMM analyses of prevalence and incidence for household contacts.

To assess the intestinal immunogenicity of mOPV2 in vaccine recipients, we examined the fraction of stools positive 1 week after the campaign in infants who received mOPV2, and we assessed one-sided hypotheses between arms (both bOPV and IPV schedules greater than tOPV; bOPV and two doses of IPV less than bOPV and one dose of IPV) for significance, with Fisher's exact test (α=0·05). We calculated viral copy numbers per g of stool using the standard curve of the MS2 extrinsic control and by normalising our Ct to the PCR efficiency of MS2, as described previously.^19^ We examined differences in the concentration of poliovirus in stool specimens positive for Sabin 2, and we ascertained significance between arms with the Wilcoxon rank-sum test for differences in the median quantity of virus shed per g of stool. We assumed missing data were missing completely at random and we excluded these data independently for each analysis. Reported sample sizes for incidence describe the number of individuals who provided at least one sample during the analysed period, and for prevalence, the number of stool samples collected. Patterns of missing data are described in the appendix (p 12).

To relate our study to historical polio transmission studies^10^, ^22^, ^23^ and knowledge of how polio vaccination regimens affect shedding and susceptibility,^24^, ^25^, ^26^ we used a dynamical model of polio transmission^25^ to explore how changes in routine immunisation schedule interact with population immunity to affect within-household transmission. We calibrated the model to data from households in which the infant received mOPV2 but household contacts did not. From measured prevalence and age-structured incidence in the 5 weeks after the mOPV2 campaign, we estimated the daily faecal-oral exposure of household contacts to infant stool and the intestinal immunities of infants and household contacts in the tOPV group and both bOPV and IPV groups combined. We then simulated within-household transmission with population immunity profiles by age characteristic of the years 2016, 2021, and 2030.

All analyses were done in *R* version 3.2.2 and MATLAB version 2015b. Additional details of analyses are provided in the appendix (pp 1–7).

This trial is registered at ClinicalTrials.gov (NCT02477046).

### Role of the funding source

The funder employs ASB and JFM, who had a role in study design, data collection, data analysis, data interpretation, and writing of the report. The corresponding author had full access to all the data in the study and had final responsibility for the decision to submit for publication.

## Results

**Figure 1:**
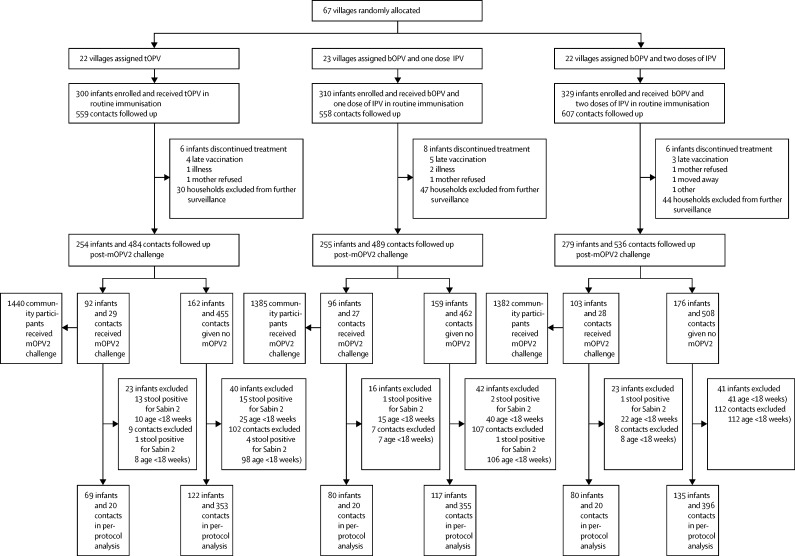
Trial profile bOPV=bivalent types 1 and 3 oral polio vaccine. IPV=inactivated polio vaccine. mOPV2=monovalent type 2 oral polio vaccine. tOPV=trivalent oral polio vaccine. Infants included in the per-protocol analysis were all children (and their household contacts) enrolled on or before Nov 1, 2015, who reached age 18 weeks by the date of the mOPV2 campaign (Jan 24, 2016) and who were not shedding Sabin 2 virus before the campaign. After the mOPV2 campaign, 788 children and their associated contacts were randomly selected from the enrolled cohort for intensive stool sampling.

At the start of the study, 67 villages were randomly allocated either tOPV (n=22), bOPV and one dose of IPV (n=23), or bOPV and two doses of IPV (n=22) as routine immunisation. Vaccines were administered according to the regimen assigned (table 1). Between April 30, 2015, and Jan 14, 2016, 939 children and 1724 household contacts were enrolled in the study (figure 1). 20 (2%) of 939 enrolled infants dropped out of the study, and 386 (3%) of 13 789 stool samples were missing from retained infants. 79 (5%) of 1724 household contacts dropped out of the study, and 2051 (8%) of 24 416 stool samples were missing from retained contacts. Table 2 shows baseline characteristics of the population and enrolment demographics (appendix p 8). Data describing baseline incidence before the mOPV2 campaign are described in the appendix (pp 8–11).

**Table 2:** Baseline characteristics

		**tOPV**	**bOPV and IPV (one dose)**	**bOPV and IPV (two doses)**
Villages (n)		22	23	22
	Population (all ages; 2012)^15^	1480 (210–4900)	990 (200–7800)	1360 (320–9700)
Enrolled infants (n)		254	255	279
	Age at enrolment (weeks)	6·4 (5·9–6·9)	6·4 (6·0–6·9)	6·4 (6·0–6·9)
	Male sex	127 (50%)	132 (52%)	144 (52%)
	Breastfeeding at enrolment	252 (99%)	250 (98%)	277 (99%)
	Age at mOPV2 campaign (weeks; per protocol)	31·7 (19·9–44·6)	30·9 (19·4–43·9)	31·4 (18·9–44·7)
	Time since last polio vaccination (weeks; per protocol)	16·1 (4·6–28·9)	15·3 (4·7–28·3)	12·3 (0·7–25·0)
	Household access to improved water	228/248 (92%)	227/253 (90%)	249/277 (90%)
	Household access to improved latrine	229/248 (92%)	230/253 (91%)	251/277 (91%)
	Maternal primary education completion*	117/248 (47%)	131/253 (52%)	162/277 (58%)
Household contacts (n)		484	489	536
	Age at enrolment (years)	15 (2·0–75)	13 (1·3–75)	19 (1·2–74)
	Younger than 5 years	79 (16%)	81 (17%)	79 (15%)
	Male sex younger than 5 years	36 (46%)	25 (31%)	35 (44%)
	Older than 17 years	235 (49%)	227 (46%)	276 (51·5%)
	Male sex older than 17 years	21 (9%)	17 (7·5%)	14 (5%)
Community participants (n)		1440	1385	1382
	Age at mOPV2 campaign (years)	2·7 (0·3–4·9)	2·7 (0·3–4·8)	2·7 (0·3–4·9)

Data are median (range) or number of corresponding population (%). Demographic attributes did not differ by study arm. bOPV=bivalent types 1 and 3 oral polio vaccine. IPV=inactivated polio vaccine. mOPV2=monovalent type 2 oral polio vaccine. tOPV=trivalent oral polio vaccine.

* Based on reported ≥9 years of schooling.

The mOPV2 campaign was carried out during Jan 25–29, 2016, and just over a third of infants were vaccinated with mOPV: 92 (36%) of 254 who received tOPV as routine immunisation, 96 (38%) of 255 who received bOPV and one dose of IPV, and 103 (37%) of 279 who received bOPV and two doses of IPV (figure 1). Furthermore, around a third of household contacts younger than 5 years received the mOPV challenge, 29 (37%) of 79 who lived in a village assigned tOPV, 27 (33%) of 81 in villages assigned bOPV and one dose of IPV, and 28 (35%) of 79 in villages allocated bOPV and two doses of IPV. Finally, 4207 community participants from households not enrolled for stool surveillance received the mOPV challenge (roughly a third of the total population younger than 5 years).

**Figure 2:**
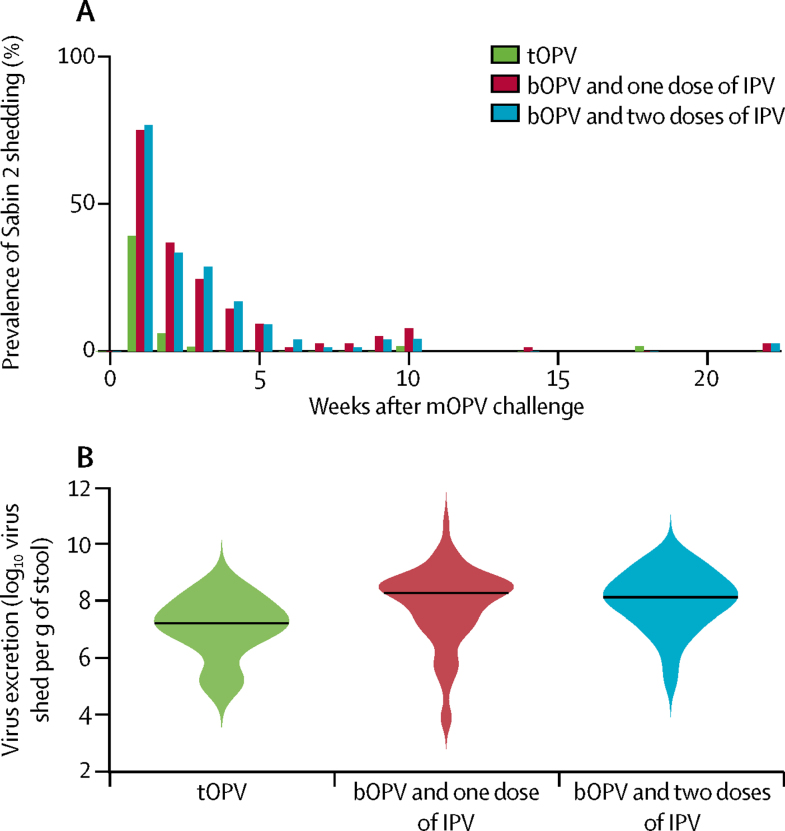
Sabin 2 shedding after mOPV2 challenge in infants who received mOPV2 bOPV=bivalent types 1 and 3 oral polio vaccine. IPV=inactivated polio vaccine. mOPV2=monovalent type 2 oral polio vaccine. tOPV=trivalent oral polio vaccine. (A) Prevalence of Sabin 2 shedding in infants who received tOPV or bOPV and IPV (one or two doses) as routine immunisation. (B) Violin plots showing the concentrations of poliovirus excreted in faeces by infants during the first 10 weeks. Black line indicates the median concentration and coloured shading the kernel density estimate of the distribution of values.

The dominant source of Sabin 2 transmission to members of the community who did not receive mOPV2 was faecal shedding from mOPV2 recipients. Infants in the combined bOPV and IPV groups who received mOPV2 shed Sabin 2 at a higher rate 1 week after the challenge than did children who received tOPV as routine immunisation (121 of 160 [76%] *vs* 27 of 69 [39%]; odds ratio [OR] 0·201, 95% CI 0·102–0·389; p<0·0001 [one-sided Fisher's exact test]; figure 2A). No difference in Sabin 2 shedding was noted between children who received one or two doses of IPV (61 of 80 [77%] *vs* 60 of 80 [75%]; OR 1·10, 95% CI 0·48–2·50; p=0·67). Of those who shed Sabin 2 virus on week 1, infants who received bOPV and one or two doses of IPV (n=124) excreted more virus than did those who received tOPV (n=29; median log_10_ copy number per g of stool, 8·2 [IQR 7·3–8·7] *vs* 7·2 [6·6–7·8]; difference 1·0, 95% CI 0·6 to 1·4; p=0·0001 [Wilcoxon rank sum test]; figure 2B). No difference in virus shed was recorded with bOPV and one (n=62) or two (n=62) doses of IPV (median log_10_ copy number per g of stool, 8·2 [IQR 7·2–8·7] *vs* 8·3 [7·5–8·7]; difference −0·1, 95% CI −0·4 to 0·3; p=0·61). Shedding duration also depended on routine immunisation. Infants who received either schedule of bOPV and IPV and were positive for Sabin 2 on day 7 shed virus for a median of 14·2 days (IQR 10·5–22·5) versus 9·5 days [6·0–14·5] with tOPV. Median durations were estimated from model maximum likelihood fits (appendix pp 13, 14). Summarising the total effect of a switch to bOPV and IPV on mOPV2 shedding in infants, the median shedding index was 15 (IQR 5–45) times higher with both schedules of bOPV and IPV combined than with tOPV.

To examine virus shedding due to mOPV2 challenge representative of the larger population of children in the community who had received tOPV in routine immunisation before the start of our study, we examined shedding in enrolled household contacts younger than 5 years who received mOPV2. As expected because of common tOPV immunisation history, no differences were noted in shedding between the combined bOPV and IPV group and tOPV group after mOPV2 challenge in these older children (ten of 35 [29%] *vs* three of 18 [17%]; OR 1·98, 95% CI 0·415–12·90; p=0·27 [Fisher's exact test]).

**Figure 3:**
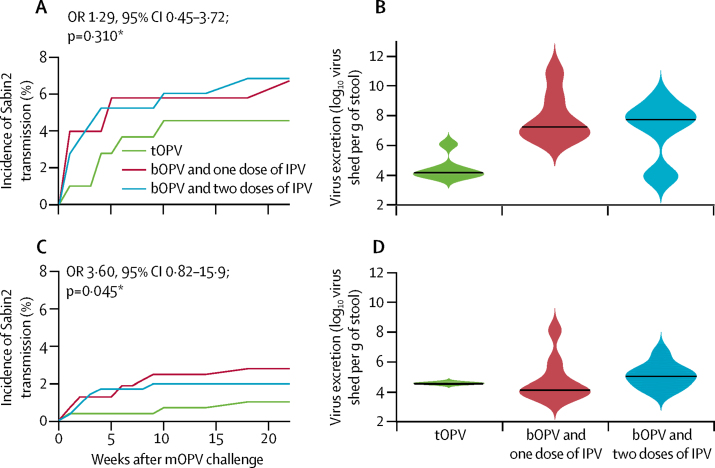
Sabin 2 shedding due to transmission in individuals who did not receive mOPV2 bOPV=bivalent types 1 and 3 oral polio vaccine. IPV=inactivated polio vaccine. mOPV2=monovalent type 2 oral polio vaccine. OR=odds ratio. tOPV=trivalent oral polio vaccine. *Difference between both bOPV and IPV groups and tOPV at 10 weeks. (A) Incidence of new poliovirus infections due to transmission in enrolled infants who received tOPV or bOPV and IPV (one or two doses) as routine immunisation. (B) Violin plots showing the concentrations of poliovirus excreted in faeces by infants who shed Sabin 2 at any point during the 10 weeks after the campaign. Black line indicates the median concentration and coloured shading the kernel density estimate of the distribution of values. (C) Incidence of new poliovirus infections due to transmission in household contacts of enrolled children who received tOPV or bOPV and IPV (one or two doses) as routine immunisation. (D) Violin plots showing the concentrations of poliovirus excreted in faeces by household contacts who shed Sabin 2 at any point during the 10 weeks after the campaign. Black line indicates the median concentration and coloured shading the kernel density estimate of the distribution of values.

Incidence of Sabin 2 acquired by transmission in enrolled infants who did not receive the mOPV2 challenge in the 10 weeks after the campaign (primary outcome) was no higher with bOPV and one or two doses of IPV relative to tOPV (15 of 252 [6%] *vs* six of 122 [4%]; OR 1·29, 95% CI 0·45–3·72; p=0·310 [Wald test for GLMM-inferred incidence after 10 weeks]; figure 3A). Mean prevalence of virus shedding over the first 10 weeks was significantly higher in the combined bOPV and IPV groups relative to tOPV (29 of 2419 stools [1%] *vs* five of 1157 stools [<1%]; OR 2·80, 95% CI 1·07–9·27; p=0·013 [Fisher's exact test]), because infants who received bOPV and either one or two doses of IPV shed for longer durations than did infants who received tOPV (median 11·5 days [IQR 7·0–21·0] *vs* 7·0 days [7·0–7·0]). Of infants who excreted Sabin 2 from transmission, those who received bOPV and one or two doses of IPV (n=15) shed significantly more virus on average than did those who received tOPV (n=6; log_10_ copy number per g of stool, median 7·7 [IQR 6·1–8·6] *vs* 4·2 [3·9–4·5]; difference 3·5, 95% CI 2·1–5·0; p=0·018 [Wilcoxon rank sum test]; figure 3B). No differences in incidence of shedding were noted with bOPV and either one or two doses of IPV (data not shown). No correlation was recorded within trial arms and no consistent trend was seen across treatment groups between transmission-acquired infection and infant age or time since last vaccination (Pearson correlations −0·12 to 0·13; p≥0·18 [Student *t* test difference from 0]).

The incidence of Sabin 2 shedding in household contacts who did not receive mOPV2 was significantly higher in contacts in the combined bOPV and IPV group relative to those in the tOPV group (17 of 751 [2%] *vs* three of 353 [1%]; OR 3·60, 95% CI 0·82–15·9; p=0·045 [Wald test for GLMM-inferred incidence after 10 weeks]; figure 3C). Prevalence exactly mirrored incidence because no contacts were recorded shedding virus for more than 1 week. As expected, because of common tOPV routine immunisation history across trial groups, no differences were noted between treatment groups in faecal concentration of virus (data not shown; figure 3D). Transmission occurred in a broad age-range of household contacts (bOPV and IPV combined group, age 3–27 years [17 contacts]; tOPV group, age 6–9 years [three contacts]; p=0·21 [two-sample Kolmogorov-Smirnov test]; appendix p 12). No significant village-level clustering of transmission-acquired infections in either infants or contacts was seen, which suggests that virus acquisition was diffuse and sporadic rather than a result of a few local transmission chains. New transmission-acquired infections were recorded up to 22 weeks after the mOPV2 campaign, 8 weeks after global tOPV cessation (figure 3A, 3C).

**Figure 4:**
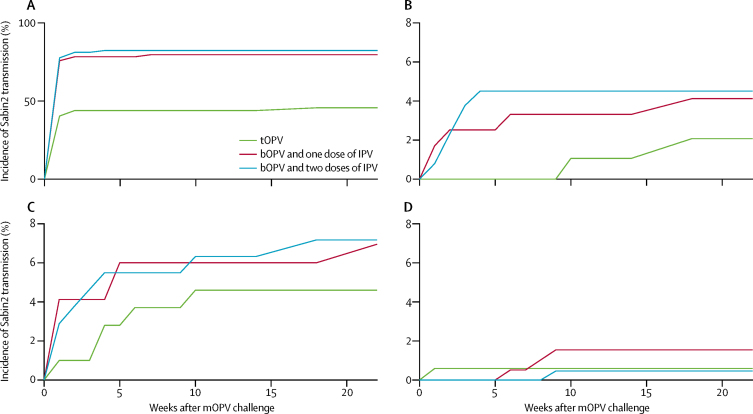
Incidence by household structure bOPV=bivalent types 1 and 3 oral polio vaccine. IPV=inactivated polio vaccine. mOPV2=monovalent type 2 oral polio vaccine. tOPV=trivalent oral polio vaccine. Incidence in households in which (A) mOPV2 was given to the infant and (B) household contacts did not receive mOPV2. Incidence in households in which mOPV2 was not given to (C) infants and (D) household contacts. Excluded from this figure are households in which at least one household contact received mOPV2.

Household incidence of virus shedding was assessed to establish the role of infants in increased shedding after mOPV2 administration and augmented susceptibility to Sabin 2 infection from transmission. Incidence of poliovirus infection due to transmission in household contacts was highest in bOPV and IPV households in which the infant received mOPV2 (household contact incidence: bOPV and IPV, ten of 277 [4%] *vs* tOPV, one of 109 [1%]; figure 4B) because of high infant incidence in the 10 weeks after vaccination (infant incidence: bOPV and IPV, 108 of 142 [76%] *vs* tOPV, 23 of 57 [40%]; figure 4A). Incidence was highest in the first 5 weeks, when infants were likely to remain shedding. In households in which no one was given mOPV2 directly, infants were more likely to be index cases (at 10 weeks: bOPV and IPV, 13 of 231 [6%] *vs* tOPV, five of 115 [4%]; figure 4C) than were older contacts (at 10 weeks: bOPV and IPV, four of 441 [1%] *vs* tOPV, one of 221 [<1%]; figure 4D). Community transmission in infants was highest with bOPV and one or two doses of IPV, reflecting the greater susceptibility of these individuals to naturally acquired infection, and no systematic differences were noted in community-acquired incidence of contacts by trial group. In both infants and contacts, incidence of Sabin 2 shedding accumulated steadily over the first 10 weeks, consistent with the transmission motif that households not receiving mOPV2 were exposed to a couple of generations of transmission from social contacts.

**Figure 5:**
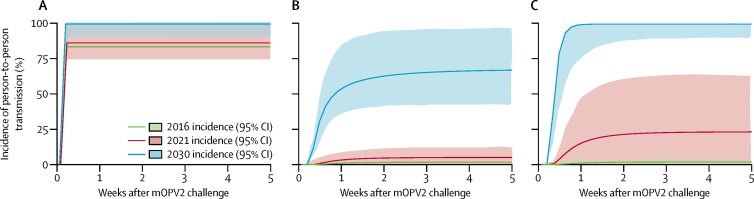
Modelled incidence in households in which the infant received mOPV2 but household contacts did not (years 2016, 2021, and 2030) Predictions of a dynamical polio model for household transmission from infants receiving mOPV2 to household contacts not receiving mOPV2. For all years, we assumed the same faecal-oral exposure from infants to household contacts, but intestinal immunity declines with time since tOPV cessation (appendix p 15). Incidence in (A) infants after receiving mOPV2, (B) enrolled household contacts (all ages), and (C) only household contacts younger than 5 years. Coloured lines depict incidence and shading indicates 95% CI. mOPV2=monovalent type 2 oral polio vaccine. tOPV=trivalent oral polio vaccine.

Figure 5 shows modelled incidence of person-to-person polio transmission within households from infants who received mOPV2 to household contacts who did not for immunity versus age profiles characteristic of the years 2016 (the two bOPV and IPV groups of this study, within 1 year of tOPV cessation), 2021 (5 years of bOPV and IPV in routine immunisation), and 2030 (bOPV and IPV to the end of 2022, then only IPV thereafter). From household prevalence and incidence data, the model inferred that intestinal immunity in infants who received bOPV and IPV in routine immunisation is lower than in our household contact population of older siblings and child-rearing adults (appendix pp 13–15) and that oral exposures of household contacts to infant stool are up to 19 μg (95% CI 3–62) of stool per day (appendix p 15).

## Discussion

In villages assigned routine immunisation with bOPV and either one or two doses of IPV, Sabin 2 shedding was highest in children vaccinated with mOPV2 and in their household contacts, but Sabin 2 transmission was not increased significantly in the community as a whole, since we did not note a pronounced rise in Sabin 2 faecal shedding in the 60% of infants who did not receive the mOPV2 challenge. Although the small observed increase in transmission does not pose an immediate threat of uncontrolled transmission of type 2 poliovirus in our well-vaccinated community, principled extrapolation from a biologically plausible polio transmission model showed that, as population immunity to Sabin 2 declines, re-introduction will be capable of seeding poliovirus transmission at intensities comparable with those seen during wild poliovirus transmission (figure 5). This intrinsically high transmission potential of Sabin 2 has much greater effect in communities in which population immunity is low. In infants who received mOPV2, we recorded an increase in Sabin 2 excretion in the combined bOPV and IPV groups compared with the tOPV group after the mOPV2 challenge. Moreover, administration of bOPV and two doses of IPV did not provide better mucosal immunity compared with bOPV and one dose of IPV. Our findings provide further evidence that, in the context of inducing primary intestinal mucosal immunity to type 2 poliovirus, the bOPV and IPV vaccine regimen is less effective compared with the previous tOPV vaccine regimen.^13^, ^14^

These findings suggest that the low intestinal immunity to Sabin 2 due to the bOPV and IPV vaccine regimen in routine immunisation, relative to tOPV in routine immunisation, decreases population immunity against Sabin 2 transmission within a short time after tOPV cessation. After completing a routine immunisation schedule of bOPV and IPV, individuals remain more susceptible to Sabin 2 infection from either mOPV2 vaccination or transmission, and those infected shed more poliovirus and drive increased transmission to contacts previously immunised with tOPV. In our well-immunised population, we recorded Sabin 2 infection attributable to the mOPV2 campaign for at least 10 weeks, and one new infection was noted 22 weeks after the campaign and, thus, 8 weeks after cessation of tOPV use in Bangladesh.

In all trial groups, transmission of Sabin 2 was limited at the community level. Household transmission typically took place within 5 weeks of the index infection (figure 4B), and community transmission lasted roughly 10 weeks (figure 4C, 4D), suggesting that only a couple of generations of transmission occurred in this well-immunised community. Our results of low baseline prevalence and sporadic transmission in rural Bangladesh support the expectation of the assured fadeout of Sabin 2 after April, 2016, in all settings with high intestinal immunity for polio irrespective of other risk factors for enteric disease transmission.

Data from the modelling study were similar to estimates previously reported in a comparable study from Houston in 1960,^10^ in which observed incidence of Sabin 2 shedding in household contacts was roughly 60% in the absence of substantial intestinal immunity.^25^ In Matlab in 2016, incidence was restricted to a few percent by immunity from tOPV completion rates of greater than 93% since 1990.^15^ As time accumulates after tOPV cessation and the age cohort with low intestinal immunity grows, household transmission after mOPV2 re-introduction will increase substantially in households with multiple children born after cessation, approaching levels seen before introduction of widespread oral polio vaccine immunisation^10^, ^23^, ^24^ by 2021, and wild poliovirus-like levels from the prevaccine era by 2030,^24^, ^25^, ^27^ with highest risk in the post-cessation birth cohort.

The main limitation of this study was the very low (<1%) baseline prevalence of Sabin 2 faecal shedding in unvaccinated infants in Matlab by comparison with published prevalences from Egypt (5%),^26^ Chile (2%),^14^ and a multicentre study in Latin America (4%).^13^ Our study results are from a rural community in Matlab, Bangladesh; therefore, they might not be directly applicable to other settings (ie, urban, less intensive health surveillance) with higher levels of baseline Sabin 2 transmission.

The small changes seen 9 months after local cessation of tOPV are potentially a harbinger of larger changes to come as birth cohorts without Sabin 2 vaccination accumulate over time. The inherent transmissibility of Sabin 2, coupled with its genetic instability and capacity to cause paralytic poliomyelitis, poses a long-term threat to polio eradication. As population immunity declines, enhanced surveillance will be necessary for timely detection and response to unexpected type 2 transmission, to prevent circulating vaccine-derived poliovirus outbreaks in addition to maintaining high coverage with IPV. Furthermore, to guarantee the long-term stability of polio eradication, it is imperative that the research community continues to develop alternative polio vaccines^2^ that have comparable intestinal immunogenicity to Sabin 2 without the risks of epidemic transmission and paralysis.
