# Supplementary material for: Community transmission of type 2 poliovirus after cessation of trivalent oral polio vaccine in Bangladesh: an open-label cluster-randomised trial and modelling study
Source: Lancet Infect Dis. 2017 Oct;17(10):1069–79. doi: 10.1016/S1473-3099(17)30358-4 (PMC5610141; doi:10.1016/S1473-3099(17)30358-4)
Supplement: Supplementary appendix [file mmc1.pdf]

THE LANCET Infectious Diseases

Supplementary webappendix

This webappendix formed part of the original submission and has been peer reviewed. We post it as supplied by the authors.

Supplement to: Taniuchi M, Famulare M, Zaman K, et al. Community transmission of type 2 poliovirus after cessation of trivalent oral polio vaccine in Bangladesh: an open-label cluster-randomised trial and modelling study. *Lancet Infect Dis* 2017; published online July 7. http://dx.doi.org/10.1016/S1473-3099(17)30358-4.

Supplementary Material

Supplemental Methods

Study design, randomization, and participants

Selection of 800 families for intensive surveillance

From the original enrolled households, we randomly selected 800 for continued intensive surveillance. We used constrained randomization implemented in MATLAB 2015b[1] to choose the retained households to mitigate two non-random features of the original enrollment: (1) that we enrolled very few contacts in the first two weeks while we amended our protocol to include the two youngest contacts instead of just contacts under age 5 years, and (2) that we had to reduce the rate of enrollment in October and November while we amended our approved protocol to permit continued bOPV+IPV vaccination after 800 children were enrolled because community acceptance and subsequent enrollment was higher than expected. The down-selection algorithm was as follows:

1) We set a weekly enrollment target that would give 800 households over the 8-month duration of the enrollment period.
2) We dropped all children with no enrolled contacts (which were uncommon after the first 4 weeks of enrollment).
3) To re-balance enrollment in time, we kept all children with at least 1 enrolled contact in any weeks that were below the weekly enrollment target.
4) For weeks with enrollment counts above the enrollment target, we randomly chose households with at least one 1 enrolled contact.

The enrolled subjects that did not receive mOPV2 provided the cohort in which we observed Sabin 2 transmission.

Outcomes

Baseline prevalence during enrollment

We also pre-specified a primary outcome to measure changes in prevalence over the enrolment period of transmission-acquired Sabin 2 infections in infant stools at 6 weeks of age, where prevalence is defined as the estimated probability that a randomly chosen infant is already shedding Sabin 2 on the day that they receive their first polio vaccination. The primary hypothesis was that prevalence would decline over time in the combined bOPV+IPV arms and thus be lower than in the tOPV arm at the end of the enrollment period. Our secondary objective was to assess if the second dose of IPV in the bOPV+2IPV arm was associated with lower prevalence of Sabin 2 in infants at the end of 9 months relative to the bOPV+1IPV arm. Additional exploratory outcomes included prevalence in household contacts, and the intestinal immunogenicity of each OPV formulation for each serotype as measured by stool shedding one day after the 6-week dose.

Statistical Analysis

Baseline prevalence in infants before the mOPV2 campaign

We estimated the prevalence in the infant per-protocol population in each arm using a mixed model in the lmer package[2] in R 3.2.2[3] to account for within-cluster correlation.

```
glmer(isPositive ~ day + day:factor(bOPV+1IPV) + day:factor(bOPV+2IPV)
   + (1|village), binomial)
```

where "isPositive" is a binary indicator describing the detection of poliovirus in the stool at the 6-week day 0 sample, "day" is the time since the trial began, "factor(bOPV+XIPV)" is the factor distinguishing the bOPV+XIPV (X=1 or 2) arm from the tOPV control arm, and "(1|village)" describes a random effect for the initial prevalence in each village. The fixed effect intercept at day equals zero is assumed to be common across study arms.   The variance of the random effects relative to the total variance estimates the intracluster correlation coefficient.[4] At low prevalence, the linear covariate with time in binomial regression represents assumed exponential decay of prevalence with time.

The statistical significance of differences between the tOPV and bOPV arms was determined by a one-sided Wald test (α=0·05) for the hypothesis that the combined mean prevalence in both bOPV+IPV arms (assuming normal residuals) is lower than the tOPV prevalence 240 days after the start of the trial, assuming common prevalence across all arms at day zero. With 800 enrolled infants, we expected at least 88% power (assuming an intra-cluster correlation coefficient of 0·05) to detect the expected decrease in Sabin 2 prevalence during enrolment in the bOPV+IPV arms, assuming a constant prevalence of 6·6% in the tOPV arm (based on data from Mirpur, Dhaka, Banglesh collected from 2010 to 2012 (M. Taniuchi, unpublished data)[5]) and falling to 0·5% by the end of enrolment in the bOPV+IPV arms (allowing for some cross-contamination from continued tOPV use in other villages).

Incidence in unvaccinated infants after the mOPV2 campaign

We estimated the incidence in the infant per-protocol population as above.

```
glmer(isInfected ~ factor(bOPV+1IPV) + factor(bOPV+2IPV) +
   (1|village), binomial)
```

where "isInfected" is a binary indicator describing if the subject tested positive for sabin 2 at any period during the ten weeks of weekly stool sampling, "factor(bOPV+XIPV)" is the factor distinguishing the bOPV+XIPV (X=1 or 2) arm from the tOPV control arm, and "(1|village)" describes a random effect for the initial prevalence in each village.

Incidence in unvaccinated household contacts after the mOPV2 campaign

We estimated the incidence in the household contact per-protocol population using a similar model.

```
glmer(isInfected ~ female + age_group + factor(bOPV+IPV) + (1|village),
    binomial)
```

where "isInfected" is a binary indicator describing whether the contact tested positive for sabin 2 at any period during the ten weeks of weekly stool sampling, "factor(bOPV+IPV)" is the factor distinguishing the combined bOPV+IPV arms from the tOPV control arm, "age_group" is a factor variable with age groups corresponding to <5 year olds, 5 to 9 years old, 10 to 17 years old and >18 years old, and "(1|village)" describes a random effect for the initial prevalence in each village.

Shedding in vaccinated infants and contacts after the mOPV2 campaign

The fraction of infants or contacts shedding OPV2 one week after direct vaccination through the mOPV2 campaign was compared using a one-sided Fisher exact test in R using the fisher.test function. Comparisons were made for: 1) combined bOPV+IPV arms vs tOPV arm, and 2) bOPV+1IPV arm vs bOPV + 2IPV arm.

Shedding index comparisons for infants and contacts after the mOPV2 campaign

For infants and contacts with stool samples collected every week for ten weeks, the mean $\log_{10}$ copy number of sabin 2 virus per gram of stool was compared using a one-sided Wilcoxon rank sum test in R using the wilcox.test function (in R this is equivalent to a one-sided Mann-Whitney test). Comparisons were made for infants and contacts between: 1) combined bOPV+IPV arms vs tOPV arm, and 2) bOPV+1IPV arm vs bOPV + 2IPV arm.

Data visualization

Prevalence was plotted as the fraction of stool samples positive for Sabin 2 each week. Incidence is shown as reverse Kaplan-Meier estimates for the cumulative probability through time that a subject becomes positive for Sabin 2. To estimate incidence while including subjects with missing stools, we assumed that subjects were not at risk during weeks when stools were not collected, such that the number at risk each week equalled the number of stools collected that week minus the number of subjects previously positive for Sabin 2.

Dynamical model of poliovirus transmission

A model of person-to-person transmission of poliovirus within households was introduced in Famulare *et al*[6] to unify the literature on the impact of intestinal immunity on poliovirus shedding and susceptibility (dose response) with detailed transmission studies to aid in reasoning about how changes in vaccine policy are likely to affect poliovirus transmission. The model is based on the premise that infants can transmit poliovirus to household contacts via fecal shedding through daily

incidental oral exposure to infant fecal matter. When infants in the model are given mOPV2, the level of infant intestinal immunity determines the probability of shedding (vaccine "take") and the fecal concentration of poliovirus and duration of shedding in those who shed. Infants who shed transmit a daily dose of poliovirus to each household contact through a daily dose of fecal matter. The probability that contacts acquire an infection is in turn determined by the dose response model and the degree of immunity possessed by the contacts. The model thus separates the roles of immunity and exposure in household transmission, and provides a rigorous basis for predicting how changing immunity would impact household transmission in settings with fixed fecal-oral exposure.

The immunity model is based on a statistical correlate of immunity—the "OPV-equivalent humoral antibody titer"—that is not directly observable. For homotypic immunity derived from live poliovirus infection (OPV or WPV), the measurable humoral antibody titer is a correlate of shedding and dose response,[7] and the OPV-equivalent antibody titer in the model is representative of the median antibody titer for cohorts of children who receive a specified routine immunization schedule.[6] For heterotypic or IPV-only immunity, where humoral antibody titer is not correlated with shedding,[7–9] the OPV-equivalent antibody titer is a hidden variable that describes the impact of vaccination on shedding and dose response in terms of equivalent OPV-based homotypic immunity.

Model specification

The full model specification with supporting data and source code is available in Famulare *et al*.[6] Here, we describe the relevant model outputs and parameters that are specific to this study. The model simulates probabilities of infection (incidence) and probabilities of shedding (prevalence) with time, assuming direct person-to-person transmission from infants ("index" therein) to household contacts ("siblings" therein); an additional transmission link in the model from household contacts to extrafamilial contacts was not used in the analyses in this paper.

Here, we denote infant prevalence in trial arm *j* with median OPV-equivalent antibody titer N_{Ab} at time *t* as $P_{jt}^{(\mathrm{inf})}(N_{Ab,j})$, and household contact prevalence with age *a* in arm *j* and titer N_{Ab} at time *t* as $P_{ajt}^{(\mathrm{con})}(N_{Ab,aj})$. All ages prevalence in household contacts is the average of the prevalence by age:

$$P_{jt}^{(\mathrm{con})} = \sum_{a=\mathrm{min\,age}}^{\mathrm{max\,age}} f_{aj}\, P_{ajt}^{(\mathrm{con})}(N_{Ab,aj}), \qquad \text{(S1)}$$

where age was binned in five year intervals and f_{aj} is the fraction of the total household contact population in age bin *a* for arm *j*. Total incidence in the household contacts by age *a* in arm *j* at 5 weeks after mOPV2 was given to the infants is denoted as $I_{aj5}^{(\mathrm{con})}(N_{Ab,aj})$.

To model immunity vs. age in the household contact population, we assumed that immunity varied with age according to the profile:

$$N_{Ab,a} = N_{Ab,<5y}(1 + (\text{age} - 30))^{-\lambda}, \quad \text{(S2)}$$

where the intercept is set by the age in children under five years of age (at bin center 30 months), $N_{Ab,<5y}$, and exponent λ determines the rate of decline. This functional form was chosen to be compatible with our model waning immunity at older ages.[6]

To model the age-dependence of fecal-oral exposure from infant to household contacts, we assumed a log-linear model of the daily fecal dose by age:

$$\log_{10}(\text{fecal exposure (grams/day)}|\text{age}) = T_{is} - \text{M}_{is}\left(\frac{\text{age} - \text{min age}}{\text{max age} - \text{min age}}\right), \quad \text{(S3)}$$

where T_{is} is the infant-sibling exposure for children under five years of age and M_{is} is the slope of the exposure vs. age relationship. We also examined a more flexible spline model but it provided no better fit and had trouble with estimation stability.

For parameter estimation, the immunity vs. age and fecal-oral exposure profiles co-vary and are not well-identified without additional constraint. In the calibration procedures described below, we assumed the immunity exponent λ and fecal-oral slope M_{is} were non-negative (and thus immunity and exposure must be constant or declining with age).

Immunity scenarios in 2016, 2021, and 2030

For our 2016 scenario, which represents the model fit to our bOPV+IPV combined trial arm, the immunity profile is given by equation S2 with parameters estimated using equations S4 and S5 as described below. For 2021, we assume that immunity for ages five years and greater obey equation S3, but that children less than five years of age receive bOPV+IPV in RI and thus immunity equivalent to that of the infants in the bOPV+IPV arm and waned for an average of 2.3 years since last RI dose at rate λ estimated with equation S2. For 2030, we assume that immunities for all ages 15 or greater obey equation S2, that all children born after 2022 receive only IPV and thus have no intestinal immunity ($N_{Ab} = 1$). Immunity in children born between 2016 and 2022 declines according to equation S3, but starting from the bOPV+IPV level, and then wanes further from 2022 to 2030, assuming no re-exposure to live poliovirus. For all years, we assumed that the fecal-oral exposure remains unchanged, given by equation S3 with parameters estimated using equation S5 (Table S4).

Model calibration

To determine the setting-specific parameters for our community in Matlab, Bangladesh that describe immunity in the infants and contacts and the inferred daily fecal-oral exposure, we calculated maximum likelihood estimates of the model from the prevalence data on shedding after mOPV2 receipt (Figure 2 A&C) and the all ages prevalence and incidence by age data for households where infants received mOPV2 and household contacts did not (Figure 4 A&B). For all immunity and

fecal-oral exposure parameters, 95% confidence intervals were estimated with 250 replicates of parametric bootstrap in which the positive detections, Y, were redrawn from the maximum likelihood prevalences, $P_j(N_{Ab,j})$, and incidences by age after 5 weeks, $I_{aj}(N_{Ab,aj})$, and the known sample counts N. All individual-level model parameters for on shedding and dose response and corresponding 95% confidence intervals were taken verbatim from Famulare *et al.*[6] For each replication, uncertainty in the individual-level model parameters was incorporated by redrawing independently from the confidence intervals assuming normality (or log-normality where appropriate).

To quantify intestinal immunities of the youngest children in Matlab—infants who received tOPV in RI, infants who received bOPV+IPV in RI, and household contacts under five years of age who received tOPV in RI prior to our study—we derived maximum likelihood estimates of the OPV-equivalent antibody titers from the data on weekly prevalence over the first 5 weeks after mOPV2 challenge. Prevalence through time after mOPV2 challenge informs immunity through shedding duration. We assumed a binomial likelihood function and independent samples each week, independent parameters for the antibody titers for each cohort (N_{Ab}), and a common setting-specific mOPV2 take multiplier (p_{S2}) that can account for study-specific differences in dose response relative to the default model:

$$\log\mathrm{L}\left(N_{Ab}, p_{S2} \middle| Y, N\right) \propto \sum_{j=1}^{3}\sum_{t=1}^{5} Y_{jt}\log\left(p_{S2}\, P_{jt}^{(\mathrm{inf})}(N_{Ab,j})\right) + (N_{jt} - Y_{jt})\log\left(1 - p_{S2}\, P_{jt}^{(\mathrm{inf})}(N_{Ab,j})\right), \qquad \text{(S4)}$$

where $j \in \{\text{tOPV infants}, \text{bOPV} + \text{IPV infants}, \text{all contacts under age 5y}\}$, and $P_{jt}^{(\mathrm{inf})}(N_{Ab,j})$ is the model prevalence at time t after mOPV2 challenge for subjects in cohort *j* with OPV-equivalent antibody titer $N_{Ab,j}$. Setting-specific take p_{S2} was assumed constant for all *j* because it is not expected to not vary by trial arm but can vary from one study to another due to nutrition, intestinal health, enterovirus burden, and detection rates between our qRT-PCR assay and the tissue culture assays on which the model was based.

Given the immunity estimates in children and the resulting response to mOPV2 challenge in infants, we derived maximum likelihood estimates of the immunity vs. age and fecal-oral exposure profiles from the prevalence and incidence data in household contacts of infants who received mOPV2, assuming a binomial likelihood function:

$$\log L(\lambda, T_{is}, M_{is} | Y_t, N_t, Y_a, N_a) \propto \sum_{j=1}^{2}\sum_{t=1}^{5} Y_{jt} \log\left(P_{jt}^{(\text{con})}\right) + \left(N_{jt} - Y_{jt}\right)\log\left(1 - P_{jt}^{(\text{con})}\right)$$

$$+\sum_{j=1}^{2}\sum_{a=\min\text{age}}^{\max\text{age}} Y_a \log\left(I_{aj5}^{(\text{con})}(N_{Ab,a})\right) + (N_a - Y_a)\log\left(1 - I_{aj5}^{(\text{con})}(N_{Ab,a})\right), \quad \text{(S5)}$$

where $j \in \{\text{tOPV infants}, \text{bOPV} + \text{IPV infants}\}$, $t \in [1,5]$ weeks, $P_{jt}^{(\text{con})}$ is the all ages prevalence, and $I_{a5}^{(\text{con})}\left(N_{Ab,a}\right)$ is the incidence after 5 weeks by age. The immunity and exposure variables are identifiable because the prevalence timeseries encodes information about shedding duration, a direct proxy of immunity, and incidence by age informs on the relative rates of transmission.

A parameter table and assessments of model fit are shown in the Supplemental Results.

Supplemental Results

Enrollment demographics

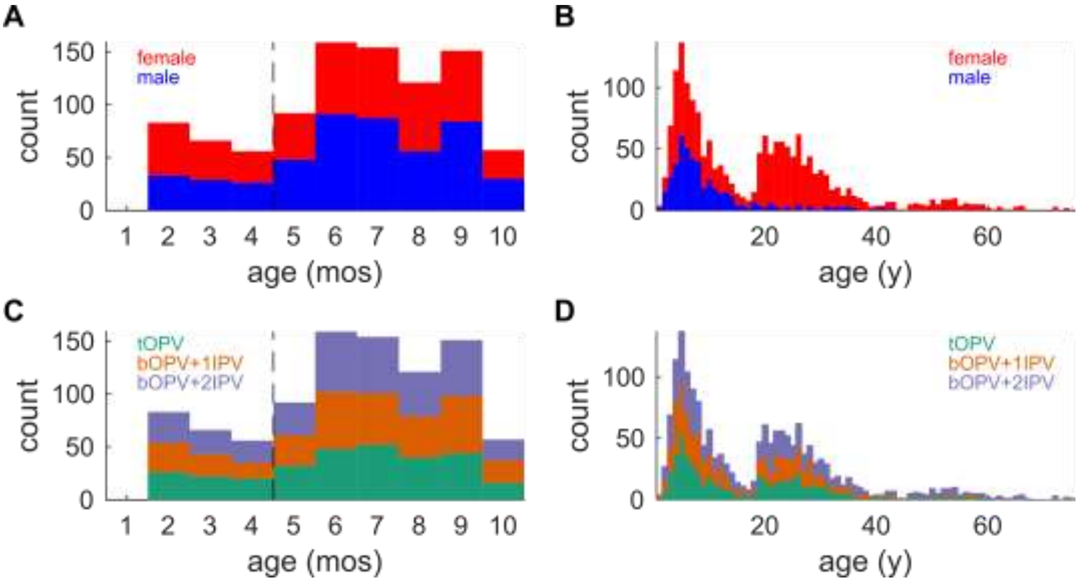

***Figure S1*: Demographic summary of enrolled infants and household contacts.** At the start of the mOPV2 campaign on 24 Jan 2016, (A) age of infants and (B) contacts by sex; (C) age of infants and (D) contacts by trial arm. Dashed line shows approximate cutoff for per-protocol population that received all scheduled routine immunizations. Through targeting for enrollment the two youngest contacts that share a cooking pot with the parent each infant, only 16% of contacts are below age 5. Most contacts above age 17 are female.

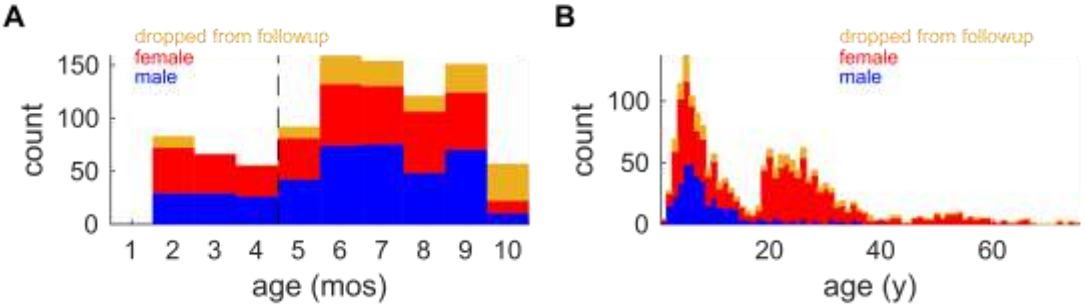

***Figure S2*: Summary of households retained for surveillance after mOPV2 challenge.** (A) Infants enrolled for followup after mOPV2 challenge. Full enrollment rates were reached on the fifth week of the study, and rates dropped during 12 October to 30 November 2015 while the enrollment protocol was being amended. 800 infants and their associated household contacts were followed for the entirety of the study. (B) Household contacts. Most households retained for intensive surveillance have two enrolled contacts.

Adverse events

292 adverse events were reported between April 30, 2015 and July 21, 2016. The most common events reported were common cold, fever, diarrhea, respiratory infections, and skin rashes. There were 196 mild, 26 moderate, and 70 severe cases. 280 of the 292 adverse events were resolved without sequelae and 10 cases were not resolved with ongoing symptoms. Two deaths were reported: one by accidental drowning and the other due to septic shock. No adverse events were associated with the vaccines used in our study.

Before the mOPV2 campaign

Sabin 2 prevalence associated with infants at 6 weeks of age.

For our estimates of the prevalence of Sabin 2 acquired by transmission at 6 weeks of age due to ongoing routine immunization, we found no significant differences between trial arms at the end of the enrollment period, and the baseline prevalence was approximately 15 times lower than our design target (Table S1). The unexpectedly low baseline prevalences in both populations left no power to detect differences by trial arm or to estimate the timescale of fadeout after tOPV cessation. Prevalence rates among household contact stools collected at the 6 week, day 0 infant timepoints (Table S2) were not significantly different from the infant prevalences (Fisher's exact tests, $p>0{\cdot}05$ for all infant-contact pairwise comparisons).

***Table S1*: Infants shedding Sabin 2 virus prior to first vaccination at 6 weeks of age.** Sample size reflects number of subjects that provided stool.

**Serotype**	**tOPV** (n=282)	**bOPV+1 IPV** (n=298)	**bOPV+2 IPV** (n=304)
Sabin 1	1 (0·4%)	1 (0·3%)	0
Sabin 2	1 (0·4%)	1 (0·3%)	2 (0·7%)
Sabin 3	1 (0·4%)	2 (0·7%)	4 (1·3%)

***Table S2*: Household contacts shedding Sabin 2 virus prior to first infant vaccination at 6 weeks of age.** Sample size reflects number of subjects that provided stool

**Serotype**	**tOPV** (n=551)	**bOPV+1 IPV** (n=555)	**bOPV+2 IPV** (n=600)
Sabin 1	0	0	0
Sabin 2	1 (0·2%)	1 (0·2%)	1 (0·2%)
Sabin 3	0	0	3 (0·5%)

tOPV and bOPV first-dose intestinal immunogenicity

Although not a stated objective of our study, the 6-week vaccination provided an opportunity to measure the intestinal immunogenicity of tOPV and bOPV in our infant cohort, as measured by poliovirus shedding in stool 24 hours after vaccination (Table S3). For Sabin 1, we see that bOPV provides significantly higher immunogenicity than tOPV (Fisher's exact test, $p<0{\cdot}0001$). In all vaccines, we found lower shedding fractions for type 3 relative to type 1 as expected.[10] In the tOPV arm, we unexpectedly saw a higher shedding fraction of Sabin 1 than Sabin 2. The unexpected low fraction of type 2 shedding after the first dose was followed by an unexpectedly high fraction of type 2 shedding four weeks after the third dose (Table S4). Together, these observations suggest that type 2 efficacy at 6 weeks of age may have been reduced by high maternal immunity against type 2[11,12] (as is also compatible with the observed low incidence due to transmission after the mOPV2 campaign), although we lack serological data to test this hypothesis. Alternatively, a serotype-specific interference from non-polio enteroviruses may also be responsible[13] (40% of n=482 children tested were positive for NPEV at 6 week, day 0 stool sample). In the bOPV arms, three of the five

Sabin 2 shedders were positive for Sabin 2 prior to vaccination, one did not pass stool the previous day but had an enrolled contact that was positive for Sabin 2, and one was negative for Sabin 2 prior to vaccination (Table S5).

***Table S3*: Positive shedders 24 hours after first vaccination.** Sample size reflects number of subjects that provided stool.

**Serotype**	**tOPV** (n=299)	**bOPV+1 IPV** (n=309)	**bOPV+2 IPV** (n=326)
Sabin 1	255 (85·2%)	289 (93·5%)	298 (91·4%)
Sabin 2	213 (71·2%)	2 (0·6%)	3 (0·9%)
Sabin 3	186 (62·2%)	185 (59·9%)	182 (55·8%)

Table S4: Positive shedders at 18 weeks of age (4 weeks after third vaccination). Sample size reflects number of subjects that provided stool.

**Serotype**	**tOPV** (n=230)	**bOPV+1 IPV** (n=242)	**bOPV+2 IPV** (n=249)
Sabin 1	14 (6·1%)	6 (2·5%)	10 (4·0%)
Sabin 2	33 (14·3%)	0	2 (0·8%)
Sabin 3	14 (6·1%)	19 (7·9%)	16 (6·4%)

Evidence for household transmission and possible persistent shedding of transmission-acquired Sabin 2

In one enrolled family (ID 551) in the bOPV+2IPV arm, both the mother and the infant were positive for Sabin 2 at the 6 week of age stool collection on September 6, 2015 (Table S5), and the infant was positive again for Sabin 2 at 18 weeks of age (December 6, 2015). The father was negative at both stool collections. We cannot determine the likely direction of transmission between mother and child due to simultaneous sample collection.

Table S5. All unambiguous transmission-acquired Sabin 2 positives. Note that for the tOPV arm, we could not distinguish the infant sample at 18 weeks of age who continued shedding from the 14 week tOPV dose and acquired Sabin 2 through transmission, and so those samples are excluded from the list. Columns S1-S3CT provide PCR cycle threshold (CT) to detection; lower CT values indicate higher viral load (detection thresholds: S1CT<36, S2CT<37, S3CT<37).

SID	Subject Type	sample type	Stool date	arm	S1CT	S2CT	S3CT
10088	contact	6 week, day 0	5/31/2015	1IPV		35·9	
330	infant	6 week, day 1*	7/24/2015	1IPV	30·2	26·5	36·0
428	infant	6 week, day 0	8/12/2015	1IPV	28·9	22·7	
74	infant	6 week, day 0	5/27/2015	2IPV		28·0	27·2
**10551**	contact	6 week, day 0	9/6/2015	2IPV		31·4	
**551**	infant	6 week, day 1**	9/7/2015	2IPV	28·6	22·0	
655	infant	6 week, day 0	9/23/2015	2IPV		28·2	
729	infant	18 week, day 0	1/14/2016	2IPV		27·3	
69	infant	6 week, day 0	5/27/2015	tOPV		24·4	
10092	contact	6 week, day 0	6/1/2015	tOPV		26·4	
10574	contact	18 week, day 0	12/10/2015	tOPV		36·9	

* infant 330 was not positive for any poliovirus the previous day.

** infant 551 did not pass stool for the 6 week, day 0 collection.

After the mOPV2 campaign

Missing stools during and after mOPV2 campaign

The fraction of subjects providing stools each week is shown in Figure S3. There were no significant differences in stool collection rates by trial arm. Stool collection rates were lowest in week 0 (pre-campaign baseline). Collection rates in week 0 were higher for subjects who received mOPV2 than those who did not (71% for infants not receiving mOPV2 vs. 90% for infants receiving mOPV2; 66% for contacts not receiving mOPV2 vs. 84% for contacts receiving mOPV2). As reported by the field research staff, missing stool samples were due to the operational challenges of both delivering mOPV2 to enrolled subjects and community participants while also collecting stool from enrolled subjects that were not to receive mOPV2. By week 2, stool collection rates stabilized for all arms and mOPV2 statuses at 96% for infants and 92% for household contacts.

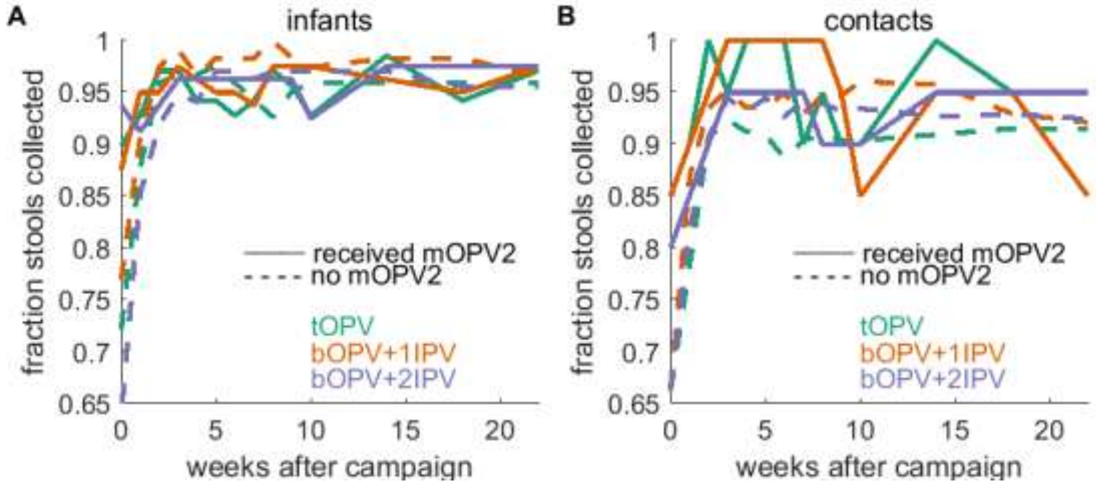

***Figure S3*: Fraction of stools collected during and after mOPV2 campaign by arm and mOPV2 treatment.** (A) Infants and (B) household contacts. Stool collection rates were lowest pre-campaign and stabilized by week 2. Within mOPV2 receipt status, there are no significant differences by trial arm.

Age distributions of contacts infected by transmission

A broader age range of the household contacts were infected by transmission in the bOPV+IPV arms.

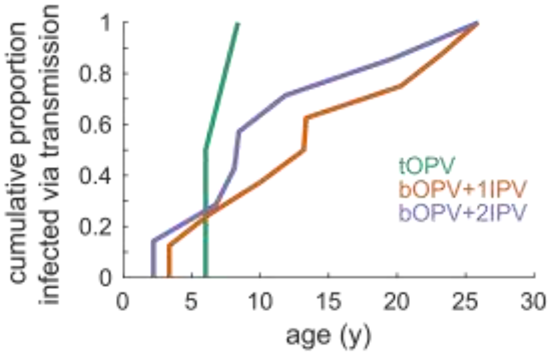

***Figure S4*. Age distributions of contacts infected by transmission.** The age range of contacts infected by transmission is wider in bOPV+IPV villages than tOPV villages. The difference is not statistically significant due to small sample sizes (tOPV, n=3; bOPV+1 IPV, n=9; bOPV+2 IPV, n=8).

Dynamical model of poliovirus transmission

The maximum likelihood estimates and 95% confidence intervals of the model parameters are shown in Table S6.

Table S6: Setting-specific parameters of household transmission model.

parameter	symbol	subject type	Value (95% CI)	Notes
Homotypic OPV-equivalent antibody titer against type 2	N_{Ab}	tOPV infant	200 (110, 310)	Inferred from prevalence by week after mOPV2 challenge (Figure S5).
		bOPV+IPV infant	31 (20, 50)	
		contacts <5y	580 (320, 1240)	
Study-specific mOPV2 take	p_{S2}	all mOPV2 recipients	1·55 (1·19, 1·97)	When the impact of intestinal immunity on dose response is accounted for, prevalence after mOPV2 challenge (take) was significantly higher than predicted by the default model parameters (which were derived from detection of poliovirus via culture[6]). The model-inferred 55% higher detection rate is consistent with the previously reported 64% higher detection rate of our qRT-PCR assay relative to tissue culture[5].
Contact immunity vs. age exponent	λ	contacts ≥5y	0·24 (0·00, 0·88)	Inferred from all ages contact prevalence and incidence by age (Figure S6), assuming immunity constant or declining with age ($\lambda \geq 0$).
Daily fecal-oral exposure between infant and household contacts	T_{is}	all contacts	-4·73 (-5·49, -4·21)	Intercept [$\log_{10}(\text{grams/day})$].
	M_{is}	all contacts	1·69 (0, 11·8)	Slope of fecal-oral exposure vs. age [$\log_{10}(\text{grams/day})$].
Individual shedding and dose response	-	all	-	Complete model specification with maximum likelihood (and 95% CI) parameters for individual fecal-oral shedding and dose response are reported in Famulare *et al*[6].

As described in the Supplemental Methods, immunity estimates in infants and children under 5 years of age were derived from our measurements of prevalence after mOPV2 challenge (Figure S5A). Our estimate of the OPV-equivalent antibody titer against type 2 was lowest for the bOPV+IPV infants and comparable to the estimates derived under the same model ($N_{Ab,bOPV+IPV} = 12\ (3{,}50)$) from a recent clinical trial reported by Asturias *et a.l*[6,9] Of those who received tOPV in RI, we observed lower immunity in infants than in the older household contacts under 5 years of age (Figure S5 B&C). As the infants had received their last dose of tOPV more recently at the time of the mOPV2 challenge, the higher intestinal immunity in the older age group is in the opposite direction expected from waning immunity. This may be due to the unexpectedly low type 2 intestinal immunogenicity of tOPV in our study as reported in Tables S1 and S2, and supplemental immunization in the older cohort (95% of whom were eligible to receive a tOPV dose in Bangladesh's last polio National Immunization Day in December 2013[14]).

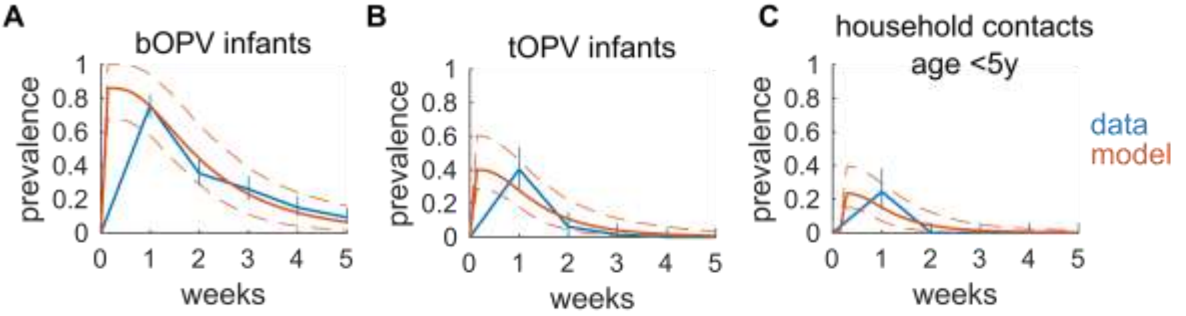

***Figure S5*: Model calibration to prevalence after mOPV2 vaccination.** Data and 95% confidence interval assuming binomial sampling (blue), model maximum likelihood fit and 95% CI (red). (A) Infants receiving tOPV in RI. (B) Infants receiving bOPV+IPV. (C) Children under five years of age born before April 30, 2015, combined across all trial arms.

Controlled for immunity, the setting-specific OPV take was 55% (19,97%) higher than would be expected from review of previous studies.[6] This likely reflects the higher detection sensitivity of our qRT-PCR assay relative to the tissue and cell culture assay data on which the model expectation was based, previously reported as 64% higher.[5]

Given the model fits to shedding after mOPV2 challenge, we fit the transmission data for household contacts of infants who received mOPV2 for the tOPV and bOPV+IPV combined arms (Figure S6).

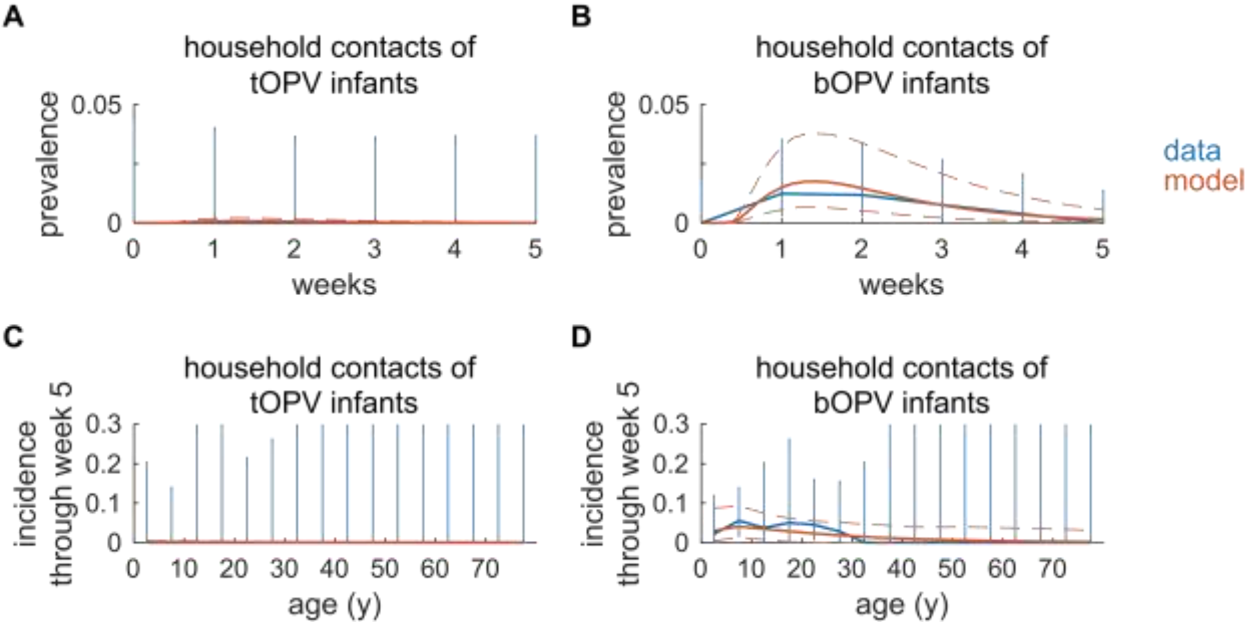

***Figure S6*: Model calibration to prevalence and incidence due to transmission.** Data and 95% confidence interval assuming binomial sampling (blue), model maximum likelihood fit and 95% CI (red). (A) Confidence intervals around zero prevalence during the first 5 weeks for all-ages prevalence in household contacts from households in the tOPV arm where the infant received mOPV2. (B) All-ages prevalence in household contacts from households in the bOPV+IPV arms where the infant received mOPV2. (C) Confidence intervals around zero incidence in household contacts through 5 weeks after the mOPV2 campaign by age for subjects in the tOPV arm. (D) Incidence in household contacts in the bOPV+IPV arms. The upper interval is greater than 0.3 (but less than 1 by definition) at higher ages due to low numbers of subjects.

The maximum likelihood fits to the bOPV+IPV combined arm provide most of the information for all parameter estimates because of the higher rate of informative positive detections; note that zero positive detections occurred during the first 5 weeks in the tOPV arm.

The fitted fecal-oral exposure and immunity vs. age profiles are shown in Figure S7. From observed transmission and quantified childhood immunity, we inferred that fecal-oral exposure was highest from infants to siblings under 5 years of age. For comparison purposes, we also show the estimated daily-fecal oral exposure between infants and siblings under 5 years of age for a comparable OPV transmission study conducted in Houston USA in 1960[15] in a population without pre-existing intestinal immunity[6]. In that study, despite incidence approaching 60% in household contacts, we estimated similar fecal-oral exposure and a lack of immunity. Together, we conclude that low incidence in Matlab is due to high immunity in the presence of moderate fecal-oral exposure.

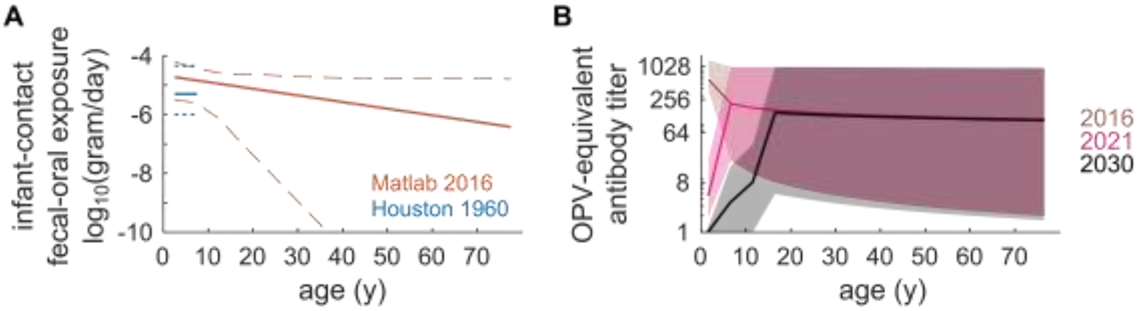

Figure S7**: Model estimates of exposure and immunity by age.** (A) Calibrated daily fecal-oral exposure of household contacts to infants by age (maximum likelihood fit and 95% CI) for Matlab (red). Shown for comparison is the same quantity estimated for 0-5 year olds in Houston in 1960[15,6] (blue). (B) Immunity profile by age under different scenarios (maximum likelihood fit and 95% CI). OPV-equivalent antibody titer is a statistical correlate of shedding and susceptibility that is inferred from shedding duration after OPV challenge as described previously.[6]

From the measured prevalence, the short shedding durations of household contacts positive for Sabin 2—rarely longer than one week—provide direct evidence of high intestinal immunity in the population across all ages. In this cohort, the maximum likelihood estimate indicates waning immunity occurs (Figure S7B), although more slowly than inferred from data collected in the Netherlands during an interval when exposure to live poliovirus (wild or vaccine) was very rare.[6,16,17] The inference of reduced waning is self-consistent with the observations from this study of transmission-acquired infections in adults.

References

1 The Mathworks: Inc. MATLAB Release 2015b. 2015.

2 Bates D, Machler M, Bolker B, *et al.* Fitting Linear Mixed-Effects Models Using {lme4}. *J Stat Softw* 2015;**67**:1–48.

3 R Core Team. R: A language and environment for statistical computing. R Foundation for Statistical Computing. 2015.

4 Nakagawa S, Schielzeth H. Repeatability for Gaussian and non-Gaussian data: A practical guide for biologists. *Biol Rev* 2010;**85**:935–56.
5 Taniuchi M, Begum S, Uddin MJ, *et al.* Kinetics of polio shedding following oral vaccination as measured by qRT-PCR versus culture. *J Clin Microbiol* 2015;**53**:206–11.
6 Famulare M, Selinger C, McCarthy KA, *et al.* Assessing the stability of polio eradication after the withdrawal of oral polio vaccine. *bioRxiv* 2017;**03/16/0840**.
7 Behrend MR, Hu H, Nigmatulina KR, *et al.* A quantitative survey of the literature on poliovirus infection and immunity. *Int J Infect Dis* 2014;**18**:4–13.
8 O'Ryan M, Bandyopadhyay AS, Villena R, *et al.* Inactivated poliovirus vaccine given alone or in a sequential schedule with bivalent oral poliovirus vaccine in Chilean infants: a randomised, controlled, open-label, phase 4, non-inferiority study. *Lancet Infect Dis* 2015;**3099**.
9 Asturias EJ, Bandyopadhyay AS, Self S, *et al.* Humoral and intestinal immunity induced by new schedules of bivalent oral poliovirus vaccine and one or two doses of inactivated poliovirus vaccine in Latin American infants: an open-label randomised controlled trial. *Lancet* 2016;**6736**:1–12.
10 Sutter RW, John TJ, Jain H, *et al.* Immunogenicity of bivalent types 1 and 3 oral poliovirus vaccine: a randomised, double-blind, controlled trial. *Lancet* 2010;**376**:1682–8.
11 World Health Organization Collaborative Study Group on Oral Poliovirus Vaccine. Factors afffecting the immunogenicity of oral poliovirus vaccine: a prospective evaluation in Brazil and the Gambia. *J Infect Dis* 1995;**171**:1097–106.
12 Patriarca PA, Wright PF, John TJ. Factors affecting the immunogenicity of oral poliovirus vaccine in developing countries: review. *Rev Infect Dis* 1991;**13**:926–39.
13 Taniuchi M, Platts-Mills JA, Begum S, *et al.* Impact of enterovirus and other enteric pathogens on oral polio and rotavirus vaccine performance in Bangladeshi infants. *Vaccine* 2016;**34**:3068–75.
14 UNICEF Bangladesh - Media centre - 21st NID to reinforce country's polio-free status. https://www.unicef.org/bangladesh/media_8593.html. 2013.
15 Benyesh-Melnick M, Melnick JL, Rawls WE, *et al.* Studies of the immunogenicity, communicability and genetic stability of oral poliovaccine administered during the winter. *Am J Epidemiol* 1967;**86**:112–36.
16 Verlinde JD, Wilterdink JB. A small-scale trial on vaccination and re-vaccination with live attenuated polioviruses in the Netherlands. In: *Preliminary Report on Mass Oral Immunization of Population Against Poliomyelitis with Live Virus from A.B. Sabin's Attenuated Strains*. 1959. 355–67.
17 Abbink F, Buisman AM, Doornbos G, *et al.* Poliovirus-specific memory immunity in seronegative elderly people does not protect against virus excretion. *J Infect Dis* 2005;**191**:990–9.
